# Frontiers and Approaches to Chemical Synthesis of Oligodeoxyribonucleotides

**DOI:** 10.3390/molecules18011063

**Published:** 2013-01-15

**Authors:** Tatyana Abramova

**Affiliations:** 1 Institute of Chemical Biology and Fundamental Medicine, Lavrent’ev Ave, 8, Novosibirsk 630090, Russia; E-Mail: abramova@niboch.nsc.ru; Tel.: +7-383-363-5183; Fax: +7-383-363-5182; 2 Laboratory of Magnetic Resonance, Scientific Research Department, Novosibirsk State University, Pirogova St., 2, Novosibirsk 630090, Russia

**Keywords:** parallel ODN synthesis, large-scale ODN synthesis, 5′-phosphorylated ODN, protective groups

## Abstract

The advantages and disadvantages of existing approaches to the synthesis of oligodeoxyribonucleotides (ODN) are discussed focusing on large-scale methods. The liquid phase and solid supported synthesis and the synthesis on soluble polymers are discussed. Different problems concerning the methods and implementation of the ODN synthesis are outlined depending on goals of using target oligomers.

## 1. Introduction

The interest in nucleic acid chemistry is due to an increasing need of synthetic oligomers of natural structure and their modified derivatives and analogs as indispensable research tools in molecular biology and fundamental medicine [1,2,3,4,5,6,7].

**Figure 1:**
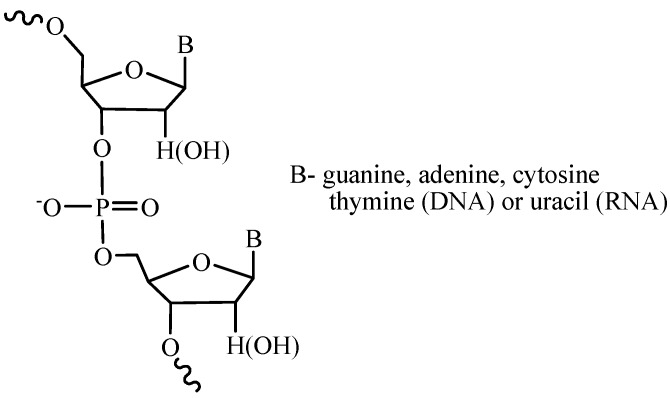
Fragment of a nucleic acid chain.

Nucleic acids are natural single- or double-stranded polymers comprising deoxyribo- or ribonucleosides linked by the phosphoric acid residues (Figure 1). The main task in the synthesis of these compounds is the binding of the monomer units in the specified order thus forming phosphodiester internucleotide bonds. In order to successfully perform this task, the phosphate group should be activated in a certain way and the functional groups, which are not involved in the reaction, should be temporarily or permanently protected.

**Figure 2:**
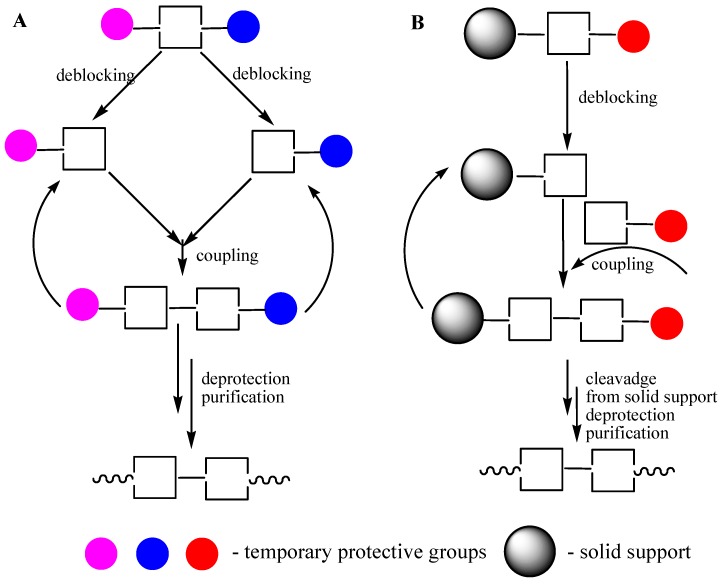
Oligomer assembling. (**A**) Block method, liquid phase synthesis (LPS). (**B**) Sequential addition of synthons, solid phase supported synthesis (SPSS).

After completion of the assembly of an oligomer by the block method or by the sequential addition of monomers (Figure 2), the removal of all protective groups and purification the product are required.

All steps of these processes must be very efficient to achieve an acceptable yield of a target oligomer. In case of the large-scale or commercial synthesis, the reaction time, reagents, and other resources are needed to be taken into consideration.

An oligonucleotide consisting of 20 monomer units may have 20^4^ various sequences. So, the synthesis of hundreds and thousands of different oligomers in small quantities (up to 1 mg of each) and the synthesis of a particular oligomer in large amount (more than 100 mg) pose quite different tasks for chemists. In this review, we will outline important aspects of the modern ODN synthesis highlighting the problems of the synthesis of both oligomers of a large variety in small qauntities and an oligomer of a certain sequence in a large amount.

## 2. Main Steps of Internucleotide Bond Formation

A comprehensive review [1] was written for the 50th anniversary of the article [8] where the first streamlined synthesis of thymidine dinucleotide of the natural structure was performed. The historically first approach was the phosphotriester method when the product of the coupling reaction between monomers was the phosphotriester derivative. However, the so-called phosphodiester approach where the product of coupling reaction was the phosphodiester derivative became more fruitful during that period [9]. ODNs synthesized by the phosphodiester approach were indispensable tools, which ensure the solution of fundamental problems of molecular biology: deciphering the genetic code [10] and proving a conceptual opportunity of the artificial gene synthesis [11,12].

Obvious limitations of the phosphodiester approach (side reactions involving phosphodiester groups) motivated scientist to the new research in the phosphotriester approach area. New phosphate protective groups [13], a step-wise polymer supported method [14], new condensing agents [15], a new phosphorylating procedure for nucleosides [16] were key points provided the wide expansion of the phosphotriester approach. For the first time, ODNs became commercially available due to the development of the phosphotriester method including both block (in the liquid phase) and sequential (in the solid phase supported) approaches (Figure 2) [17].

**Figure 3:**
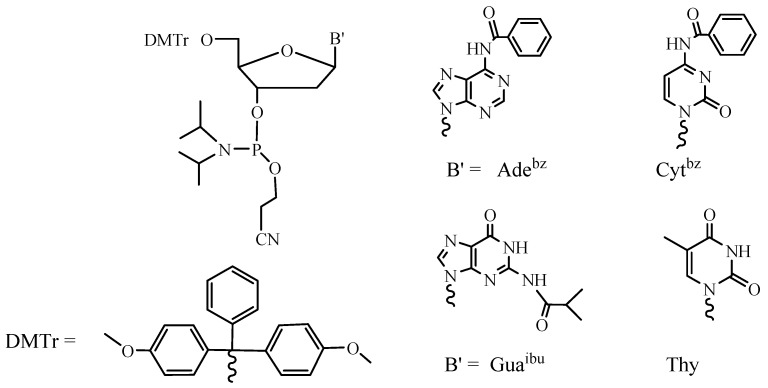
Standard phosphoramidites used in ODN SPSS.

It is easy to understand that the block approach can be performed in the liquid phase and the sequential approach is more beneficial while using the solid support. A growing oligonucleotide chain is covalently linked to the solid support in the SPSS approach, which facilitates the removal of the excess of reagents and allows for the automation of the whole process consisting of the repeating steps. Due to the heterogeneous media, the reactivity of the activated nucleotide derivative is of particular importance in this case. Therefore, nucleotide synthons containing more active P(III) groups are now exclusively used in SPSS of oligonucleotides. Application of phosphoramidites [18] (Figure 3) and *H*-phosphonates [19] led to the fact that the synthesis of oligonucleotides became a routine procedure and provided for the wide commercial availability of these compounds and their analogs and derivatives [20].

Thus far, many effective methods have been developed for oligonucleotide bond formation. The application of these methods is closely related to the approaches (LPS or SPSS) and strategies (block coupling or sequential addition of monomers) used in the ODN synthesis. ODNs with the length of up to 100 units and in the amount of up to 1 µmol obtained by the phosphoramidite SPSS are commercially available. To obtain larger amounts of oligonucleotides (>10 µmol of a purified compound), it is necessary to improve the effectiveness of the overall process by increasing the yields of certain stages, minimizing the excess of reagents, and developing more efficient methods for purification of target products. Moreover, the engineering of new oligonucleotide synthesizers is an indispensible milestone for these projects. Most of these problems have been solved [21], but large-scale synthesis is not still widely commercialized.

## 3. Problems of Modern ODN Synthesis

### 3.1. Fast Oligomer Deprotection

As mentioned above, the synthesis of hundreds and thousands of different oligomers in small amounts (up to 1 mg of each) poses particular challenges. The first one is the minimization of the time needed for the synthesis and the treatment of an oligomer. Now the synthesis of one ODN takes 1.5–2 h using modern DNA synthesizers and the phosphoramidite SPSS approach (3–5 min for one elongation cycle). At the same time, deprotection of the product takes 16 h in aqueous ammonia at 55 °C in the case of the standard protective groups for heterocyclic bases (Figure 3).

**Figure 4:**
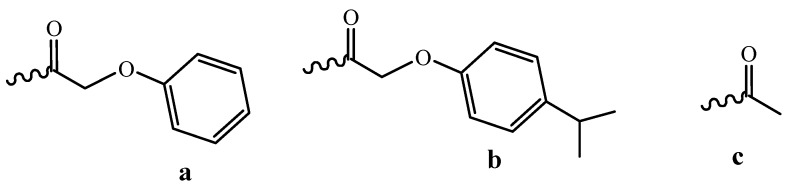
Labile protective groups for nucleoside heterocyclic bases: (**a**) phenoxyacetyl, (**b**) p-isopropylphenoxyacetyl, (**c**) acetyl.

Two ways for decreasing the time of this process are possible: the use of more labile protective groups and the development of new deprorection protocols. Nowadays, the most popular among the labile protective groups are the phenoxyacetyl group for adenine, the *p*-isopropylphenoxyacetyl group for guanine and the acetyl group for cytosine (Figure 4) [22].

It takes 2 h to remove these groups. This approach is very useful when ODNs bearing unstable moieties such as cyanine or rodamine dyes must be synthesized [23]. Reduced storage stability of synthons (Figure 4) and their solutions prevents the wide use of such phosphoramidites in the routine synthesis.

To remove the standard protective groups, the ethanolamine treatment has been proposed [24], which causes no transamination of cytosine. Deprotection in this case takes 1.5 h. It is possible to reduce this time to 5 min but for this purpose the acetyl protective group for cytosine is needed to avoid transamination [25].

One more possibility of fast deprotection of ODNs is using thermolabile protective groups (TPG) for phosphate and hydroxyl centers based on 2-pyridyl assisted cyclization [26,27]. A new “click-clack” approach valuable for obtaining biologically important phosphate esters and their analogs and some *H*-phosphonate derivatives is developed [28]. This approach further contributes in controlling thermolabile properties of TPG groups.

### 3.2. High-Throughput Parallel Synthesis of ODNs

At the beginning of the 21st century, the parallel synthesis of individual ODNs became another way of decreasing the synthesis time. To implement this strategy, multichannel DNA synthesizers were developed. [29]. Most of the instruments are now constructed to simultaneously produce 96 ODNs [30]. Moreover, an economical 2 × 96 synthesizer was described [31]. In this paper, the problem of the quality of synthesized ODNs is considered, which is of great importance because only spot checking is possible when hundreds and thousands of ODNs are obtained daily.

DNA synthesizer for parallel synthesis of 1536 ODNs was later developed [32]. The careful design of reaction cells, optimization of flow systems, and the choice of materials contacting with a solid support allowed for a high reliability of ODN synthesis avoiding truncated sequences and modified nucleosides.

**Figure 5:**
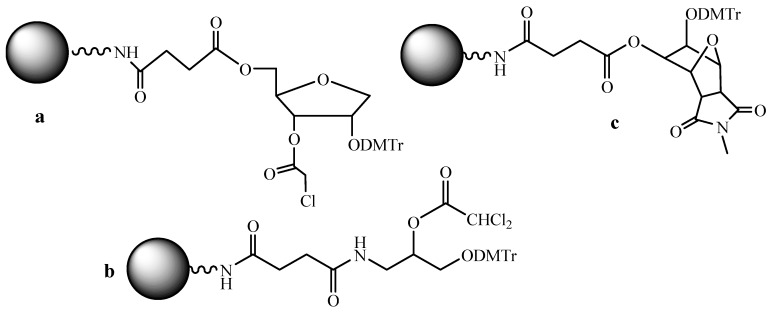
Universal supports for ODN synthesis.

Among the specific problems associated with parallel SPSS of ODNs, it is worth noting the correct choice of the support loaded with the first nucleoside unit. Usually, manufactures propose the supports with a pre-loaded nucleoside that is at least four kinds of supports. In the case of the multichannel synthesis it would, obviously, be rational to unify the support in such a way that to use in all reaction cells the same support to exclude the mistakes of the choice of the first nucleoside. Various linkers mostly containing two vicinal hydroxyl groups were described (Figure 5) [33,34] for this purpose.

In the paper [35], six variants of commercially available universal supports of types **a** and **b** (Figure 5) were compared. The highest yield was obtained when using the support of type **b**. In this case, the milder conditions for the cleavage of an ODN from the polymer support are required due to the nearby amide group. Acceleration of the cleavage can also be achieved due to the rigid spatial structure of the universal linker type **c** (Figure 5) [36].

### 3.3. Preparative ODN Synthesis

Alongside the efficient parallel synthesis of thousands of different ODNs, instruments for the preparative synthesis of ODN of a unique sequence were designed. As noted earlier [21], the whole manufacturing technology should be submitted to FDA for approval any preparation as a drug. So, the design of the large-scale ODN synthesizers is an urgent task. The authors of review [37] mention that there are two main approaches to engineering such apparatus. First, scaling of the existing synthetic protocols and simultaneous optimization of chemical procedures should be performed. A simple increase of reaction volumes without changing conditions rarely succeeds. In the second approach, engineers deeply transform the design of reaction vessels and the flow system of an apparatus in order to obtain maximum yields [37].

**Figure 6:**
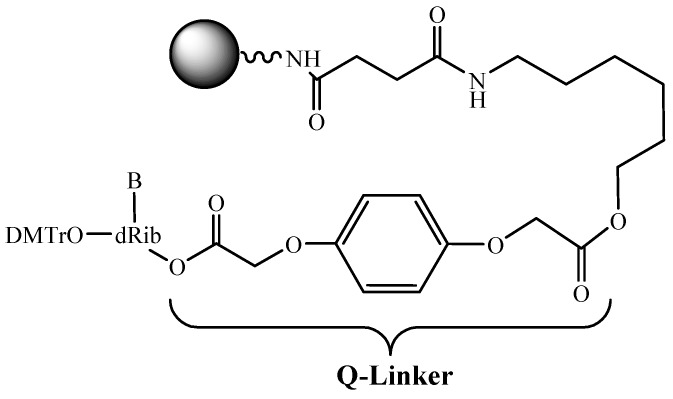
Reusable support for ODN synthesis.

As noted above, the preparative ODN synthesis implies special attention to the cost effectiveness of the production. A multiple usage of the support can further contribute to the solution of this problem. For this purpose, a new linker group for the attachment of a first nucleoside to the support is proposed [38] (Figure 6). Up to six ODN synthesis was performed while using this linker arm on the commercially available LCAA-CPG.

Other important moments are the finding of a new inexpensive, safe and effective catalyst, pyridinium trifluoriacetate, for the synthesis of phosphoramidite synthons [39] and a new coupling stage in SPSS phophoramidite method for the formation of the internucleiotide bonds [40], and the excluding of highly toxic and hard recyclable solvents [41]. The search of new sulfurization reagents for obtaining the most popular as antisense agents phosphorothioate ODN analogs [42] is also a valuable task.

While small amounts of ODNs for diagnostic purposes, as a rule, does not require purification, oligomers, which are planned to be used as drugs should be purified. Furthermore, the preparation of products of the standard quality at minimum cost and the ease of scaling are of great importance in this case. Various chromatographic and extraction processes were proposed for the purification of tens of grams of ODNs of natural structure and their phosphorothioate analogs [43,44,45].

### 3.4. ODN Synthesis in Liquid Phase

**Figure 7:**
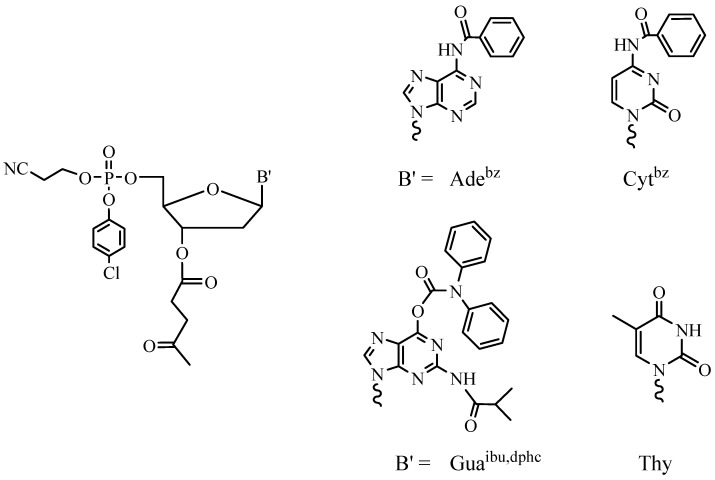
5′-Phosphorylated synthons used in the phosphotriester synthesis of 5′-prosphoryated ODNs.

As noted above, the phosphotriester method for the ODN synthesis using both the LPS and SPSS approaches provided for the first time commercially available ODNs. In our opinion, LPS has not lost its significance, especially in the synthesis of short ODNs (up to 16 units) by the phosphotriester method on the preparative scale. Although this process is time- and labor-consuming, it still has advantages: the virtual absence of restrictions on the scale of the synthesis and the relative cost-effectiveness since it does not require using a large (10–30-fold) excess of reagents. It is worthwhile noting that not only 5′-hydroxyl containing ODNs [46] but also 5′-phosphorylated oligomers [47,48,49] can be obtained by the phosphotriester block LPS approach without additional phosphorylation steps. The 5′-phosphate group is a convenient site for obtaining ODN conjugates [50] due to the well-developed methods of selective activation of the mono-esterified phosphate group [51]. Moreover, this group is essential for ligase-mediated reaction in genetic engineering. In order to obtain 5′-phosphorylated ODNs by the phosphotriester approach, fully protected 5′-phosphorylated synthons shown in Figure 7 [52] should be used instead of usually utilized 5′-*O*-DMTr 3′-phophorylated monomers. The purification of the target 5′-phosphorylated ONDs could be more efficient in the case of block approach (Figure 2) usually used jointly with LPS due to the greater difference in length of parent and target oligomers [48,52].

**Figure 8:**
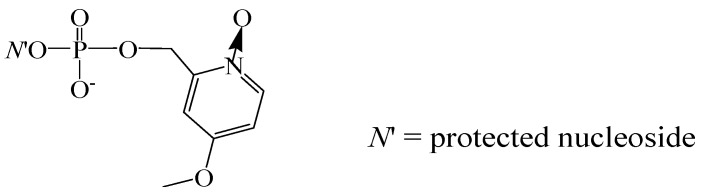
4-Methoxy-1-oxido-2-picolyl protective group.

A new impetus to the improvement of the phosphotriester block synthesis in liquid phase was given when new catalytically active phosphate protective groups were proposed [53,54]. With the use of the 4-methoxy-1-oxido-2-picolyl group (Figure 8), a number of natural DNA and RNA oligomers and their phosphorothioate, *C*-phosphonate, and 2′-*O*-methylated derivatives were successfully synthesized [55,56]. This approach was proposed as an alternative to the SPSS phophoramidite method for the synthesis of RNA oligonucleotides [57].

**Figure 9:**
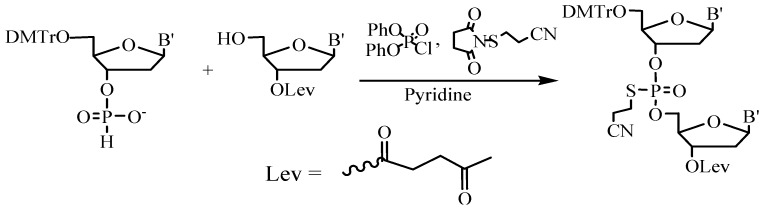
Four-component *H*-phopsphonate phosphorothioate ODN synthesis.

An interesting example of the preparative liquid phase block synthesis of phosphorothioate ODN Vitravene^TM^ based on the modified *H*-phosphonate approach was described [58]. To increase the stability of the internucleotide *H*-phosphonate phosphodiester group and to achieve high yields, the oxidation of the newly formed internucleoside bonds was carried out simultaneously with their protection (Figure 9).

New protocols proposed for LPS of ODN are often aimed at simplifying the overall procedure because one of the disadvantages of LPS is increased labor intensiveness. For example, the authors of [59] succeeded in the synthesis of a hexamer omitting chromatographic purification of intermediate oligomers. The liquid-phase trinucleosides preparation based on various methods for the coupling of monomeric units (phosphotriester, phosphite triester, phosphoramidite) is currently the most widely used approach to obtaining synthons for the synthesis of mixed oligonucleotides necessary for random mutagenesis [60].

### 3.5. Soluble Supports in ODN Synthesis

The advantages of SPSS over LPS are obvious: it is much less laborious and can easily be automated. On the other hand, heterogeneous reactions are, as a rule, less efficient and require the higher excess of reagents and prolonged time of the treatment. The approach combining the advantages of both SPSS and LPS seems to be an excellent alternative to these methods (HELP-method) [61]. The authors proposed the use of monomethyl ether of polyethylene glycol (MPEG) of an average molecular weight of 5,000 Da as a support for the oligomer synthesis. This substance is soluble in methylene chloride and pyridine but readily crystallizes upon addition of diethyl ether. So, the synthetic reactions (for example, coupling) can be carried out in solution, followed by the precipitation and washing of the support with a bound growing oligonucleotide chain. The HELP approach can be applied in combination with the phosphoramidite method for the synthesis of natural ODNs [62] and their phosphorothioate analogs, particularly, in the preparative scale [63]. New materials such as protected β-cyclodextrin are now being developed as the soluble support for the ODN synthesis [64].

An alternative to the growing oligomer chain on the soluble support, it is possible to use reagents immobilized on soluble supports, e.g., polymer-bound activators of the coupling reaction [65,66].

## 4. Conclusions

This minireview considered the major problems of the modern ODN synthesis. At the early development stages of the ODN synthesis, the progress in the nucleic acid chemistry gave a powerful impetus to the development of the fundamental research in molecular biology [9,10]. Now, it seems like the achievements in molecular biology and fundamental medicine are the force for progress in the ODN synthesis, e.g., the rapid development of the parallel ODN synthesis may be explained by the appearance of new methodologies and applications in molecular biology in the beginning of 90th (PCR, modern sequencing methods) and, as a result, a great need in a large number of various primers. In the last 20 years, significant efforts were made to automate the large-scale processes and increase an efficiency of the synthesis in order to meet the needs of fundamental research in medicine (antisense and therapeutic ODNs).

Currently, the main effort in the ODN synthesis is focused on improving production technology of DNA chips for diagnostics [67].Production of DNA chips is very economical, but the amount of ODN, which can be obtained in this way is very small (about 10 fmol) [68] and a quality of the products is not sufficient for PCR. At the same time, about 10 nmol of the oligomer of a good quality can be obtained using a modern high throughput ODN synthesizer. To fill the gap between these scales while maintaining efficiency of the synthesis and providing the needed amount of a product of good quality, the new microfluidic ODN synthesizers are being developed [69,70,71]. Such apparatus allow obtaining 10–1000 pmol of ODNs and subsequent successful ligase-mediated assembly of dsDNA of 200 b.p without preliminary purification and amplification of resulting fragments [72].

Unfortunately, despite many years of clinical trials of more than 40 different natural ODNs and their analogues, only one substance (Vitravene^TM^) has been approved to date as a therapeutic agent [4]. This fact undoubtedly retards the development and implementation of large-scale (1–100 kg) production of ODNs and their analogs. Based on the available data, it is impossible to give a preference to any of the proposed approaches to the ODN synthesis: SPSS, LPS, or the synthesis using the soluble support. As mentioned in review [1], the combination of these approaches may be the best choice, e.g., the synthesis of short blocks (tetramers, hexamers, *etc*.) in liquid phase followed by SPSS. Further studies are topical in this area because in any case efficient processes and apparatuses are indispensable for successful implementation of pharmaceutical programs.
